# The complete mitochondrial genome of the chocolate chip sea cucumber *Isostichopus badionotus* (Echinodermata: Holothuroidea)

**DOI:** 10.1080/23802359.2021.1937365

**Published:** 2021-06-14

**Authors:** Victoria I. Drake, Erica Kim, Hailee G. Nigro, Viktoria E. Bogantes, Alexis M. Janosik

**Affiliations:** Chemistry, University of West Florida, Pensacola, FL, USA

**Keywords:** Cookie dough sea cucumber, *Isostichopus badionotus*, Holothuroidea, mitogenome, mtDNA

## Abstract

The chocolate chip sea cucumber, *Isostichopus badionotus* (Selenka 1867), is an ecologically and biomedically important species. In this study, we report the complete mitogenome sequence of the sea cucumber, *I. badionotus* (Echinodermata: Holothuroidea). The mitochondrial genome consisted of 16,319 bp, with 13 protein-coding genes, 22 tRNA genes, and 2 rRNA genes. The total nucleotide composition consisted of 31.61% A, 29.20% T, 23.48% C, 15.71% G, with a high A + T content of 60.81%. Phylogenetic analysis using the complete mitochondrial genome of *I. badionotus* is helpful in studying the evolution of beneficial adaptations to aid in bioremediation and biomedical research and development.

*Isostichopus badionotus* (Selenka, 1867) commonly known as the chocolate chip sea cucumber is an important marine species both ecologically and biomedically. In particular, *I. badionotus* is a key bioremediator as a sediment feeder excreting non-organic waste. Specifically, *I. badionotus* prevents the suffocation of higher trophic levels by repressing excess organic matter (Purcell et al. 2016). However, natural populations of *I. badionotus* are declining due to rising commercial value and codependency in fishing economies. Contemporary studies have suggested overexploitation by industries near Colombia, Cuba, and Mexico (Slater et al. 2018; López-Rocha and Velázquez-Abunader 2019). In terms of biomedical importance, sea cucumbers contain potential anti-inflammatory bioactive components in their body-wall, and some studies advocate viable use of *I. badionotus* in anticancer compounds (Pérez-Espadas 2014; Olivera-Castillo et al. 2020).

While the most common variation is fully dark brown, *I. badionotus* is known for the morphotype with the eponymous coloration of light brown with dark brown to black spots. *Isostichopus badionotus* was reclassified into the order Synallactida in 2017 when the order Aspidochirotida, previously the most diverse amongst the Holothurians, was deemed polyphyletic (Miller et al. 2017). The complete mitochondrial genome is an excellent resource for studying the evolution of favorable traits and adaptations within a family. Here, we report the complete mitochondrial genome of *I. badionotus*, which will provide useful genetic data for studying molecular ecology and biomedical applications, as well as for species identification.

The specimen of *I. badionotus* was collected from St. Andrews Bay at the St. Andrews State Park jetties in Bay County, FL (30°07'25.1″N, 85°43'57.8″W). The specimen is deposited in the invertebrate collection at Florida Museum of Natural History (www.floridamuseum.ufl.edu, John D. Slapcinsky, slapcin@flmnh.ufl.edu) under voucher number Echinodermata 021830. Total genomic DNA was extracted using the QIAGEN DNeasy Blood and Tissue Kit (Qiagen, Valencia, CA) following the manufacturer’s protocol. DNA libraries were constructed using Illumina HiSeq (Illumina, San Diego, CA), and were sequenced using HiSeq platform, with 250-bp paired-end reads at the Hubbard Center for Genomics, Sequencing Core Facility (Durham, NH). Phred quality score of 30 and minimum read length of 150 base-pairs. The DNA fragments were assembled using *de novo* assembly method in Geneious Prime V. 2021.0.3 (https://www.geneious.com). To infer phylogenetic placement, a maximum-likelihood phylogenetic tree was constructed using MEGA-X (Kumar et al. 2018) with 1000 bootstrap replicates (Figure 1). Mitochondrial genome sequences used for this tree were: *Apostichopus japonicus*, EU294194; *Apostichopus nigripunctatus*, NC_013432; *Apostichopus parvimensis*, NC_029699; *Apostichopus californicus*, NC_026727; *Stichopus monotuberculatus*, MN276189; *Stichopus chloronotus*, MW218897; *Stichopus* sp. SF-2010, HM853683; *Stichopus horrens*, MN128376; *Synallactes* sp. Y30071, MT559281. Annotation of the assembled genome was conducted with MITOS2 (Bernt et al. 2013).

**Figure 1.**
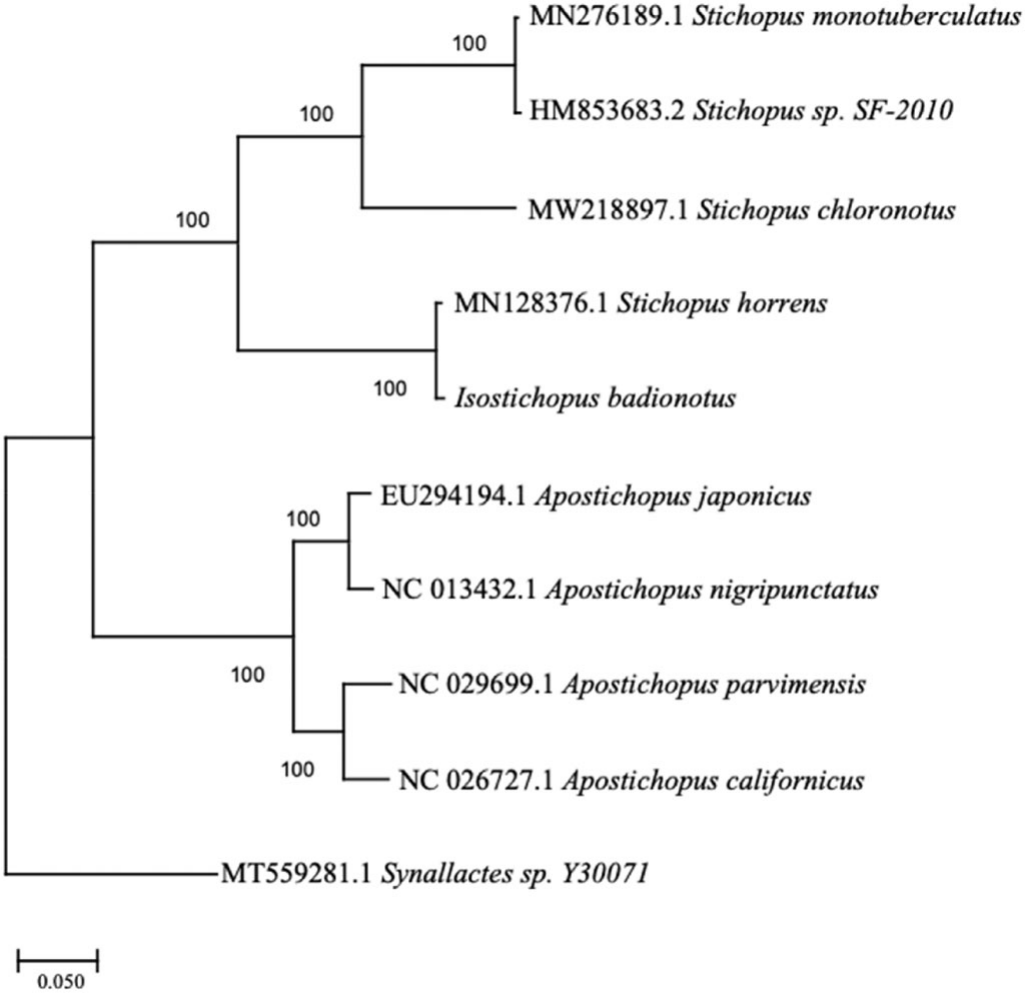
The phylogenetic relationship of *Isostichopus badionotus* based on the full mitochondrial genomes eight species in the family *Stichopodidae,* with *Synallactes* sp. as the outgroup.

The complete mitogenome of the *I. badionotus* was 16,319 bp in length (GenBank accession number: MZ188901) and contained 13 protein-coding genes, 22 tRNA and two rRNA genes, as found in other holothurian mitogenomes. The base composition of the mitogenome was estimated to be 31.61% A, 29.20% T, 23.48% C, 15.71% G, with an A + T content of 60.81%. The A + T content is similar to but slightly higher than *Stichopus horrens* (16,315bp, 60.74% A + T)*. Isostichopus badionotus* clustered with *S. horrens*, making the *Stichopus* genus polyphyletic. Additional mitochondrial genome sequencing of closely related species is necessary to further resolve phylogenetic relationships within the family Stichopodidae. The complete mitochondrial genome of *I. badionotus* is beneficial in studying the evolution of beneficial adaptations to aid in biomedical research and further phylogenetic research of Stichopodidae and in related families.

## Data Availability

The data that support the findings are openly available in NCBI at (https://www.ncbi.nlm.nih.gov/), reference number (MZ188901). The associated BioProject, SRA, and Bio-Sample numbers are PRJNA731158, SRR14596717, and SAMN19272657.
